# Supplementary material for: Omicron BA.2 (B.1.1.529.2): high potential to becoming the next dominating variant
Source: ArXiv. 2022 Feb 10:arXiv:2202.05031v1. Preprint. [Version 1] (PMC8845508)
Supplement: 1 [file NIHPP2202.05031V1-supplement-1.pdf]

Supporting information for Omicron BA.2 (B.1.1.529.2): high potential to becoming the next dominating variant

Jiahui Chen[1] and Guo-Wei Wei[1,3,4*]
[1] Department of Mathematics,
Michigan State University, MI 48824, USA.
[2] Department of Electrical and Computer Engineering,
Michigan State University, MI 48824, USA.
[3] Department of Biochemistry and Molecular Biology,
Michigan State University, MI 48824, USA.

February 11, 2022

S1 Supplementary figures

Figure S1 provides the statistic analysis of BFE changes of RBD-ACE2 induced by mutations from Alpha, Beta, Gamma, Lambda, and Mu.

S2 Supplementary data

The Supplementary_Data.zip contains four files as listed in the following subsection.

S2.1 Disrupted antibodies

File antibodies_BFEs.csv shows the BFE changes of antibodies disrupted by Omicron mutations.

S2.2 List of antibodies

File antibodies.csv lists the Protein Data Bank (PDB) IDs for all of the 185 SARS-CoV-2 antibodies.

S3 Supplementary feature generation methods

In this section, the workflow of the deep learning-based BFE change predictions of protein-protein interactions induced by mutations for the present SARS-CoV-2 variant analysis and prediction will be firstly introduced, which includes four steps as shown in Figure S2: (1) training data preparation; (2) feature generations of protein-protein interaction complexes; and (3) prediction of protein-protein interactions by deep neural networks. Next, the validation of our machine learning-based model will be demonstrated, suggesting consistent and reliable results compared to the experimental deep mutations data.

*Corresponding author. Email: weig@msu.edu
*Corresponding author. Email: weig@msu.edu

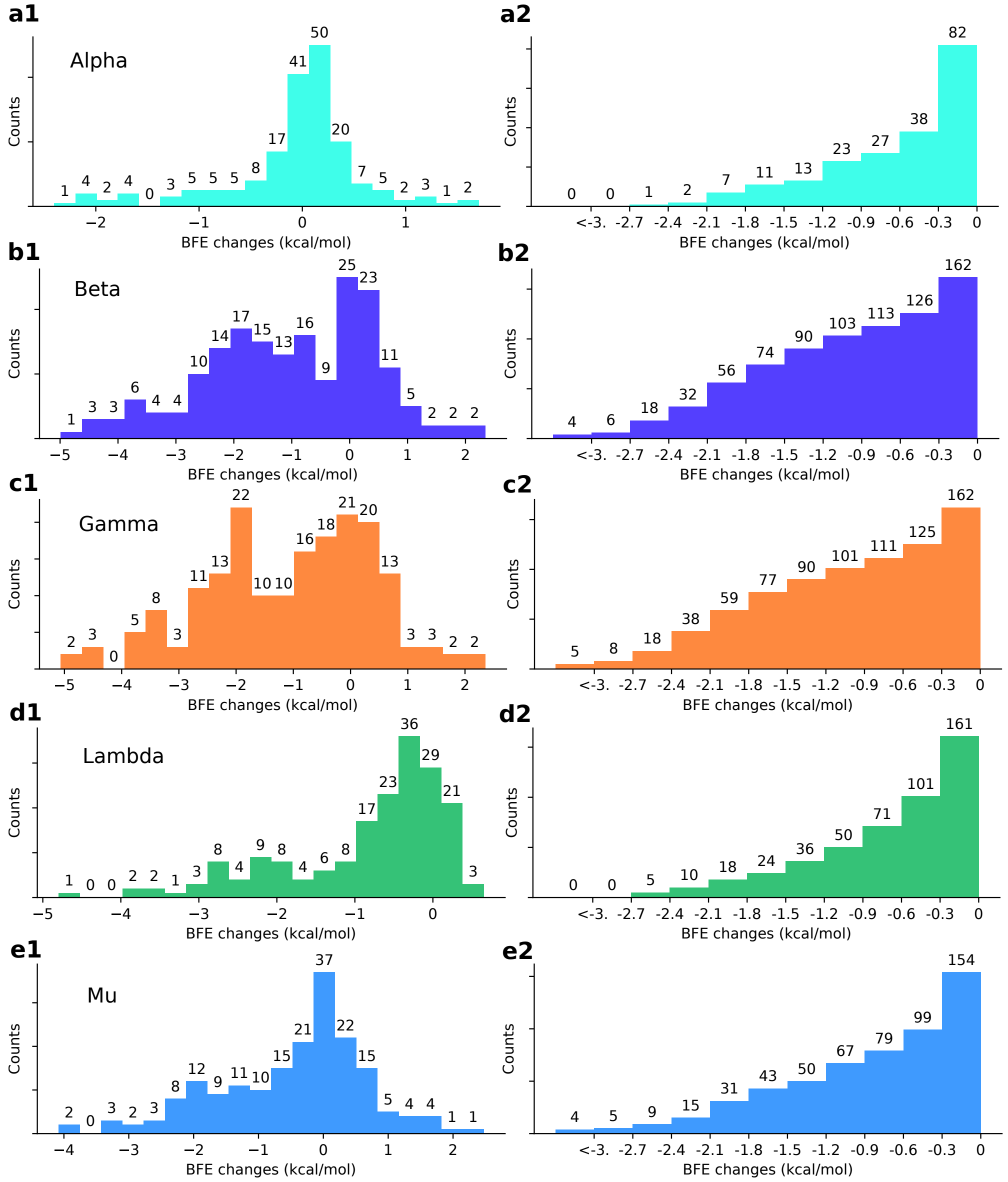

Figure S1: The extension of Figure 3. Analysis of variant mutation-induced BFE changes of 185 antibody-RBD complexes. **a1, b1, c1, d1** and **e1** The distributions (counts) of accumulated BFE changes induced by Alpha, Beta, Gamma, Lambda, and Mu mutations respectively for 185 antibody-RBD complexes. For each case, there are more mutation-weakened complexes than mutation-strengthened complexes. **a2, b2, c2, d2** and **e2** The numbers of antibody-RBD complexes regarded as disrupted by Alpha, Beta, Gamma, Lambda, and Mu mutations respectively under different thresholds ranging from 0 kcal/mol, -0.3 kcal/mol, to <-3 kcal/mol.

S3.1 Methods for BFE change predictions

In this section, the process of the machine learning-based BFE change predictions is introduced. Once the data pre-processing and SNP genotyping are carried out, we will firstly proceed with the training data preparation process, which plays a key role in reliability and accuracy. A library of 185 antibodies and RBD complexes, as well as an ACE2-RBD complex, are obtained from Protein Data Bank (PDB). RBD mutation-induced BFE changes of these complexes are evaluated by the following machine learning model. According to the emergency and the rapid change of RNA virus, it is rare to have massive experimental BFE change data of SARS-CoV-2, while, on the other hand, next-generation sequencing data is relatively easy to collect. In the training process, the dataset of BFE changes induced by mutations of the SKEMPI 2.0

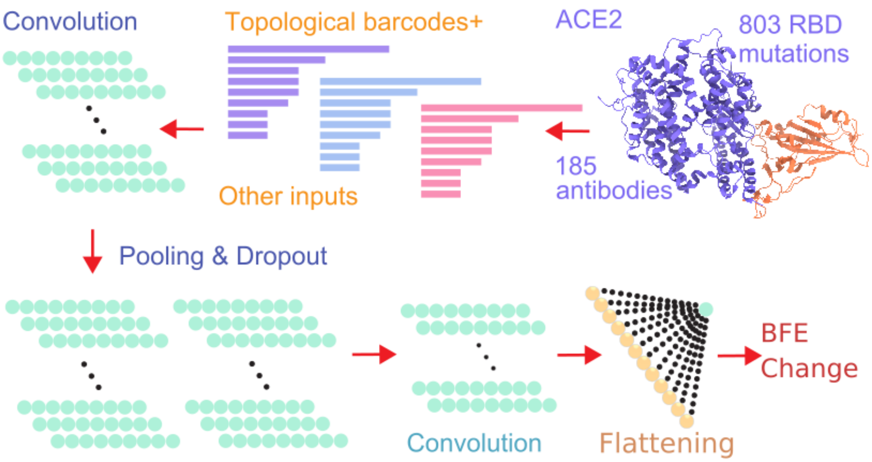

Figure S2: Illustration of genome sequence data pre-processing and BFE change predictions.

dataset [1] is used as the basic training set, while next-generation sequencing datasets are added as assistant training sets. The SKEMPI 2.0 contains 7,085 single- and multi-point mutations and 4,169 elements of that in 319 different protein complexes used for the machine learning model training. The mutational scanning data consists of experimental data of the binding of ACE2 and RBD induced mutations on ACE2 [2] and RBD [3, 4], and the binding of CTC-445.2 and RBD with mutations on both protein [4].

Next, the feature generations of protein-protein interaction complexes are performed. The element-specific algebraic topological analysis on complex structures is implemented to generate topological bar codes [5–8]. In addition, biochemistry and biophysics features such as Coulomb interactions, surface areas, electrostatics, et al., are combined with topological features [9]. The detailed information about the topology-based models will be demonstrated in subsection S3.2. Lastly, deep neural networks for SARS-CoV-2 are constructed for the BFE change prediction of protein-protein interactions [5]. The detailed descriptions of dataset and machine learning model are found in the literature [5, 10, 11] and are available at TopNetmAb.

S3.2 Feature generation for machine learning model

S3.2.1 Topology features

Among all features generated for machine learning prediction, the application of topology theory makes the model to a whole new level. Those summarized as other inputs are called as auxiliary features and are described in Section S3.3.2 and S3.3.3. In this section, a brief introduction about the theory of topology will be discussed. Algebraic topology [6, 7] has achieved tremendous success in many fields including biochemical and biophysical properties [8]. Special treatment should be implemented for biology applications to describe element types and amino acids in poly-peptide mathematically, which element-specific and site-specific persistent homology [10, 12]. To construct the algebraic topological features on protein-protein interaction model, a series of element subsets for complex structures should be defined, which considers atoms from the mutation sites, atoms in the neighborhood of the mutation site within a certain distance, atoms from antibody binding site, atoms from antigen binding site, and atoms in the system that belong to type of {C, N, O}, $\mathcal{A}_{\rm ele}$(E). Under the element/site-specific construction, simplicial complexes is constructed on point clouds formed by atoms. For example, a set of independent $k+1$ points is from one element/site-specific set $U = \{u_0, u_1, ..., u_k\}$. The k-simplex σ is a convex hull of $k+1$ independent points U, which is a convex combination of independent points. For example, a 0-simplex is a point and a 1-simplex is an edge. Thus, a m-face of the k-simplex with $m+1$ vertices forms a convex hull in a lower dimension $m < k$ and is a subset

of the $k+1$ vertices of a k-simplex, so that a sum of all its $(k-1)$–faces is the boundary of a k–simplex σ as

$$\partial_k \sigma = \sum_{i=1}^{k} (-1)^i \langle u_0, ..., \hat{u}_i, ..., u_k \rangle, \tag{3}$$

where $\langle u_0, ..., \hat{u}_i, ..., u_k \rangle$ consists of all vertices of σ excluding u_i. The collection of finitely many simplices is a simplicial complex. In the model, the Vietoris-Rips (VR) complex (if and only if $\mathbb{B}(u_{i_j}, r) \cap \mathbb{B}(u_{i_{j'}}, r) \neq \emptyset$ for $j, j' \in [0, k]$) is for dimension 0 topology, and alpha complex (if and only if $\cap_{u_{i_j} \in \sigma} \mathbb{B}(u_{i_j}, r) \neq \emptyset$) is for point cloud of dimensions 1 and 2 topology [8].

The k-chain c_k of a simplicial complex K is a formal sum of the k-simplices in K, which is $c_k = \sum \alpha_i \sigma_i$, where α_i is coefficients and is chosen to be $\mathbb{Z}_2$. Thus, the boundary operator on a k-chain c_k is

$$\partial_k c_k = \sum \alpha_i \partial_k \sigma_i, \tag{4}$$

such that $\partial_k : C_k \to C_{k-1}$ and follows from that boundaries are boundaryless $\partial_{k-1}\partial_k = \emptyset$. A chain complex is

$$\cdots \xrightarrow{\partial_{i+1}} C_i(K) \xrightarrow{\partial_i} C_{i-1}(K) \xrightarrow{\partial_{i-1}} \cdots \xrightarrow{\partial_2} C_1(K) \xrightarrow{\partial_1} C_0(K) \xrightarrow{\partial_0} 0, \tag{5}$$

as a sequence of complexes by boundary maps. Therefore, the Betti numbers are given as the ranks of kth homology group H_k as $\beta_k = \text{rank}(H_k)$, where $H_k = Z_k/B_k$, k-cycle group Z_k and the k-boundary group B_k. The Betti numbers are the key for topological features, where β_0 gives the number of connected components, such as number of atoms, β_1 is the number of cycles in the complex structure, and β_2 illustrates the number of cavities. This presents abstract properties of the 3D structure.

Finally, only one simplicial complex couldn't give the whole picture of the protein-protein interaction structure. A filtration of a topology space is needed to extract more properties. A filtration is a nested sequence such that

$$\emptyset = K_0 \subseteq K_1 \subseteq \cdots \subseteq K_m = K. \tag{6}$$

Each element of the sequence could generate the Betti numbers $\{\beta_0, \beta_1, \beta_2\}$ and consequentially, a series of Betti numbers in three dimensions is constructed and applied to be the topological fingerprints in Figure S2.

S3.2.2 Residue-level features

Mutation site neighborhood amino acid composition Neighbor residues are the residues within 10 Å of the mutation site. Distances between residues are calculated based on residue C_α atoms. Six categories of amino acid residues are counted, which are hydrophobic, polar, positively charged, negatively charged, special cases, and pharmacophore changes. The count and percentage of the 6 amino acid groups in the neighbor site are regrading as the environment composition features of the mutation site. The sum, average, and variance of residue volumes, surface areas, weights, and hydropathy scores are used but only the sum of charges is included.

pKa shifts The pKa values are calculated by the PROPKA software [13], namely the values of 7 ionizable amino acids, namely, ASP, GLU, ARG, LYS, HIS, CYS, and TYR. The maximum, minimum, sum, the sum of absolute values, and the minimum of the absolute value of total pKa shifts are calculated. We also consider the difference of pKa values between a wild type and its mutant. Additionally, the sum and the sum of the absolute value of pKa shifts based on ionizable amino acid groups are included.

Position-specific scoring matrix (PSSM) Features are computed from the conservation scores in the position-specific scoring matrix of the mutation site for the wild type and the mutant as well as their difference. The conservation scores are generated by PSI-BLAST [14].

Secondary structure The SPIDER2 software is used to compute the probability scores for residue torsion angle and residues being in a coil, alpha helix, and beta strand based on the sequences for the wild type and the mutant [15].

S3.2.3 Atom-level features

Seven groups of atom types, including C, N, O, S, H, all heavy atoms, and all atoms, are considered when generating the element-type features. Meanwhile, other three atom types, i.e., mutation site atoms, all heavy atoms, and all atoms, are used when generating the general atom-level features.

Surface areas Atom-level solvent excluded surface areas are computed by ESES [16].

Partial changes Partial change of each atom is generated by pdb2pqr software [17] using the Amber force field [18] for wild type and CHARMM force field [19] for mutant. The sum of the partial charges and the sum of absolute values of partial charges for each atomic group are collected.

Atomic pairwise interaction interactions Coulomb energy of the ith single atom is calculated as the sum of pairwise coulomb energy with every other atom as

$$C_i = \sum_{j,j\neq i} k_e \frac{q_i q_j}{r_{ij}}, \tag{7}$$

where k_e is the Coulomb's constant, r_{ij} is the distance of ith atom to jth atom, and q_i is the charge of ith atom. The van der Waals energy of the ith atom is modeled as the sum of pairwise Lennard-Jones potentials with other atoms as

$$V_i = \sum_{j,j\neq i} \epsilon \Big[\big(\frac{r_i + r_j}{r_{ij}}\big)^{12} - 2\big(\frac{r_i + r_j}{r_{ij}}\big)^{6} \Big], \tag{8}$$

where ϵ is the depth of the potential well, and r_i is van der Waals radii.

In atomic pairwise interaction, 5 groups (C, N, O, S, and all heavy atoms) are counted both for Coulomb interaction energy and van der Waals interaction energy.

Electrostatic solvation free energy Electrostatic solvation free energy of each atom is calculated using the Poisson-Boltzmann equation via MIBPB [20] and are summed up by atom groups.

S4 Supplementary machine learning methods

The topology-based network model for BFE change predictions induced mutations on SARS-CoV-2 studying applies a deep neural network structure. Similar approaches have been widely implemented in the energy prediction of protein-ligand binding [21] and protein-protein interactions [12]. The neural network method maps an input feature layer to output layer and mimics biological brains for solving problems where numerous neuron units are involved and weights of neurons are updated by backpropagation methods. To make more complicated structure in order to extract abstract properties, more layers and more neurons in each layer can be constructed. In the training process, optimization methods are applied to avoid overfitting issue, such as dropout methods [22] that a partial of computed neurons of each layer is dropped. For the model cross validations, the Pearson correlation of 10-fold cross-validation is 0.864, and the root mean square error is 1.019 kcal/mol.

S4.1 Deep learning algorithms

A deep neural network is a neural network method with multi-layers (hidden layers) of neurons between the input and output layers. In each layer, the single neuron gets fully connected with the neurons in the next layer. It should preserve the consistency of all labels when applying the model for mutation-induced BFE change predictions. The loss function is constructed as follows:

$$\operatorname*{argmin}_{W,b} L(W,b) = \operatorname*{argmin}_{W,b} \frac{1}{2}\sum_{i=1}^{N} \big(y_i - f(x_i;\{W,b\})\big)^2 + \lambda \|W\|^2 \tag{9}$$

where N is the number of samples, f is a function of the feature vector x_i parameterized by a weight vector W and bias term b, and λ represents a penalty constant.

S4.2 Optimization

The backpropagation is applied to evaluate the loss function starting from the output layer and propagates backward through the network structure to update the weight vector W and bias term b. Since gradient calculation is required, therefore, we apply the stochastic gradient descent method with momentum, which only evaluates a small part of training data and can be considered as calculating exponentially weighted averages, which is given as

$$\begin{aligned} V_i &= \beta V_{i-1} + \eta \nabla_{W_i} L(W_i, b_i) \\ W_{i+1} &= W_i - V_i, \end{aligned} \tag{10}$$

where W_i is the parameters in the network, $L(W_i, b_i)$ is the objective function, η is the learning rate, X and y are the input and target of the training set, and $\beta \in [0, 1]$ is a scalar coefficient for the momentum term. The momentum term involved accelerates the converging speed.

S5 Supplementary validation

In the main content, we briefly summarized validations of our machine learning predictions and experimental data. For large quantitative validations, we compared the BFE change prediction for mutations on S protein RBD to the experimental deep mutational enrichment data on RBD binding to human ACE2 and CTC-445.2 induced by RBD mutations [4, 5, 9]. To make these validations, we eliminated the experimental deep mutational enrichment data of RBD binding to human ACE2 and CTC-445.2 from the training sets and set them as testing sets, which have 1539 and 1500 samples, respectively. In the validation of RBD and CTC-445.2 complex, there is a very high correlation between the enrichment data and machine learning predictions, as well as the validation of RBD binding to ACE2, with Pearson correlations are 0.69 and 0.70, respectively. The deep mutational enrichment data can give a proportional descriptor of the affinity strength of protein-protein interactions induced by mutations. The machine learning methods, however, give stable and equalized evaluations, while experimental data might be different dramatically due to conditions and environments.

In addition, we compared our machine learning results with other experimental data, which are escape fraction, pseudovirus infection changes, and IC_{50} fold changes [5]. In the comparison of 35 cases to experimental escape fractions on RBD binding to clinical trial antibodies induced by emerging mutations, our machine learning predictions have a Pearson correlation of 0.80. Especially, those high escaping mutations E484K and E484Q on LY-CoV555, and mutations K417T and K417N on LY-CoV016, are indicated by both our predictions and the experimental data [5]. We also use the pattern comparisons of our prediction to experimental data. Lastly, we collected experimental data from different literature [23–26]. According to variations from different research groups, they were summarized in increasing/decreasing patterns of emerging variant (including co-mutations) impacts on antibody therapies in clinical trials. In total, there are 20 pattern comparisons with an excellent agreement between various experimental data and our predictions, except for a minor discrepancy [5].

References

[1] Justina Jankauskaitė, Brian Jiménez-García, Justas Dapkūnas, Juan Fernández-Recio, and Iain H Moal. SKEMPI 2.0: an updated benchmark of changes in protein–protein binding energy, kinetics and thermodynamics upon mutation. *Bioinformatics*, 35(3):462–469, 2019.

[2] Erik Procko. The sequence of human ace2 is suboptimal for binding the s spike protein of sars coronavirus 2. *BioRxiv*, 2020.

[3] Tyler N Starr, Allison J Greaney, Sarah K Hilton, Daniel Ellis, Katharine HD Crawford, Adam S Dingens, Mary Jane Navarro, John E Bowen, M Alejandra Tortorici, Alexandra C Walls, et al. Deep mutational scanning of SARS-CoV-2 receptor binding domain reveals constraints on folding and ACE2 binding. *Cell*, 182(5):1295–1310, 2020.

[4] Thomas W Linsky, Renan Vergara, Nuria Codina, Jorgen W Nelson, Matthew J Walker, Wen Su, Christopher O Barnes, Tien-Ying Hsiang, Katharina Esser-Nobis, Kevin Yu, et al. De novo design of potent and resilient hACE2 decoys to neutralize SARS-CoV-2. *Science*, 370(6521):1208–1214, 2020.

[5] Jiahui Chen, Kaifu Gao, Rui Wang, and Guo-Wei Wei. Revealing the threat of emerging SARS-CoV-2 mutations to antibody therapies. *Journal of Molecular Biology*, 433(7744), 2021.

[6] Gunnar Carlsson. Topology and data. *Bulletin of the American Mathematical Society*, 46(2):255–308, 2009.

[7] Herbert Edelsbrunner, David Letscher, and Afra Zomorodian. Topological persistence and simplification. In *Proceedings 41st annual symposium on foundations of computer science*, pages 454–463. IEEE, 2000.

[8] Kelin Xia and Guo-Wei Wei. Persistent homology analysis of protein structure, flexibility, and folding. *International journal for numerical methods in biomedical engineering*, 30(8):814–844, 2014.

[9] Jiahui Chen, Kaifu Gao, Rui Wang, and Guo-Wei Wei. Prediction and mitigation of mutation threats to COVID-19 vaccines and antibody therapies. *Chemical Science*, 12(20):6929–6948, 2021.

[10] Jiahui Chen, Rui Wang, Menglun Wang, and Guo-Wei Wei. Mutations strengthened SARS-CoV-2 infectivity. *Journal of molecular biology*, 432(19):5212–5226, 2020.

[11] Rui Wang, Yuta Hozumi, Changchuan Yin, and Guo-Wei Wei. Mutations on COVID-19 diagnostic targets. *Genomics*, 112(6):5204–5213, 2020.

[12] Menglun Wang, Zixuan Cang, and Guo-Wei Wei. A topology-based network tree for the prediction of protein–protein binding affinity changes following mutation. *Nature Machine Intelligence*, 2(2):116–123, 2020.

[13] Delphine C Bas, David M Rogers, and Jan H Jensen. Very fast prediction and rationalization of pka values for protein–ligand complexes. *Proteins: Structure, Function, and Bioinformatics*, 73(3):765–783, 2008.

[14] Stephen F Altschul, Thomas L Madden, Alejandro A Schäffer, Jinghui Zhang, Zheng Zhang, Webb Miller, and David J Lipman. Gapped blast and psi-blast: a new generation of protein database search programs. *Nucleic acids research*, 25(17):3389–3402, 1997.

[15] Yuedong Yang, Rhys Heffernan, Kuldip Paliwal, James Lyons, Abdollah Dehzangi, Alok Sharma, Jihua Wang, Abdul Sattar, and Yaoqi Zhou. Spider2: A package to predict secondary structure, accessible surface area, and main-chain torsional angles by deep neural networks. In *Prediction of protein secondary structure*, pages 55–63. Springer, 2017.

[16] Beibei Liu, Bao Wang, Rundong Zhao, Yiying Tong, and Guo-Wei Wei. Eses: software for e ulerian solvent excluded surface, 2017.

[17] Todd J Dolinsky, Jens E Nielsen, J Andrew McCammon, and Nathan A Baker. Pdb2pqr: an automated pipeline for the setup of poisson–boltzmann electrostatics calculations. *Nucleic acids research*, 32(suppl_2):W665–W667, 2004.

[18] David A Case, Tom A Darden, Thomas E Cheatham, Carlos L Simmerling, Junmei Wang, Robert E Duke, Ray Luo, MRCW Crowley, Ross C Walker, Wei Zhang, et al. Amber 10. Technical report, University of California, 2008.

[19] Bernard R Brooks, Charles L Brooks III, Alexander D Mackerell Jr, Lennart Nilsson, Robert J Petrella, Benoît Roux, Youngdo Won, Georgios Archontis, Christian Bartels, Stefan Boresch, et al. Charmm: the biomolecular simulation program. *Journal of computational chemistry*, 30(10):1545–1614, 2009.

[20] Duan Chen, Zhan Chen, Changjun Chen, Weihua Geng, and Guo-Wei Wei. Mibpb: a software package for electrostatic analysis. *Journal of computational chemistry*, 32(4):756–770, 2011.

[21] Duc Duy Nguyen and Guo-Wei Wei. AGL-Score: Algebraic graph learning score for protein–ligand binding scoring, ranking, docking, and screening. *Journal of chemical information and modeling*, 59(7):3291–3304, 2019.

[22] Nitish Srivastava, Geoffrey Hinton, Alex Krizhevsky, Ilya Sutskever, and Ruslan Salakhutdinov. Dropout: a simple way to prevent neural networks from overfitting. *The journal of machine learning research*, 15(1):1929–1958, 2014.

[23] FACT SHEET FOR HEALTH CARE PROVIDERS EMERGENCY USE AUTHORIZATION (EUA) OF REGEN-COV (fda.gov).

[24] Yiska Weisblum, Fabian Schmidt, Fengwen Zhang, Justin DaSilva, Daniel Poston, Julio CC Lorenzi, Frauke Muecksch, Magdalena Rutkowska, Hans-Heinrich Hoffmann, Eleftherios Michailidis, et al. Escape from neutralizing antibodies by SARS-CoV-2 spike protein variants. *Elife*, 9:e61312, 2020.

[25] Pengfei Wang, Manoj S Nair, Lihong Liu, Sho Iketani, Yang Luo, Yicheng Guo, Maple Wang, Jian Yu, Baoshan Zhang, Peter D Kwong, et al. Antibody resistance of SARS-CoV-2 variants B. 1.351 and B. 1.1. 7. *Nature*, 10, 2021.

[26] Delphine Planas, David Veyer, Artem Baidaliuk, Isabelle Staropoli, Florence Guivel-Benhassine, Maaran Michael Rajah, Cyril Planchais, Françoise Porrot, Nicolas Robillard, Julien Puech, et al. Reduced sensitivity of SARS-CoV-2 variant delta to antibody neutralization. *Nature*, pages 1–7, 2021.
